# What do primary care physicians and researchers consider the most important patient safety improvement strategies?

**DOI:** 10.1186/1472-6963-11-102

**Published:** 2011-05-16

**Authors:** Sander Gaal, Wim Verstappen, Michel Wensing

**Affiliations:** 1 Scientific Institute for Quality of Healthcare, Radboud University Nijmegen Medical Centre, Nijmegen, the Netherlands

## Abstract

**Background:**

Although it has been increasingly recognised that patient safety in primary care is important, little is known about the feasibility and effectiveness of different strategies to improve patient safety in primary care. In this study, we aimed to identify the most important strategies by consulting an international panel of primary care physicians and researchers.

**Methods:**

A web-based survey was undertaken in an international panel of 58 individuals from eight countries with a strong primary care system. The questionnaire consisted of 38 strategies to improve patient safety. We asked the respondents whether these strategies were currently used in their own country, and whether they felt them to be important.

**Results:**

Most of the 38 presented strategies were seen as important by a majority of the participants, but the use of strategies in daily practice varied widely. Strategies that yielded the highest scores (>70%) regarding importance included a good medical record system (82% felt this was very important, while 83% said it was implemented in more than half of the practices), good telephone access (71% importance, 83% implementation), standards for record keeping (75% importance, 62% implementation), learning culture (74% importance, 10% implementation), vocational training on patient safety for GPs (81% importance, 24% implementation) and the presence of a patient safety guideline (81% importance, 15% implementation).

**Conclusion:**

An international panel of primary care physicians and researchers felt that many different strategies to improve patient safety were important. Highly important strategies with poor implementation included a culture that is positive for patient safety, education on patient safety for physicians, and the presence of a patient safety guideline.

## Background

Patient safety is receiving increased attention worldwide [1]. In the last decades, the focus of patient safety research has been mostly focused on hospital care, [2] although in recent years patient safety in primary care has been evolving as well. This is an important development, as most patients attain their health care in primary care settings, particularly in countries with a strong primary care system [3]. Various definitions of patient safety have been published, [4] and probably the shortest description is 'to do no harm to patients'. Primary care has been found to be relatively safe, although incidents with major consequences occur in this setting as well [4-6].

In primary care practice, strategies to improve patient safety may be based on reporting and analysis of incidents or they may target specific high risk domains, such as medication safety [7]. The scope of patient safety in primary care was perceived by physicians and nurses to be very broad [8]. In the context of Linneaus (see http://www.linneaus-pc.eu), an international study on patient safety in primary care, physicians and researchers with an interest in patient safety were asked what they considered to be important approaches to improve patient safety in primary care. Our aim was to document the perceived importance and current use of a range of strategies in order to guide future research and development in this field.

## Methods

### Study design and setting

A web-based survey was conducted in a convenience sample of mostly European primary care physicians and researchers with an interest in patient safety. These were recruited in eight countries with a relatively strong primary care system: Austria, Denmark, France, Germany, the Netherlands, New Zealand, Slovenia and the United Kingdom. We identified a key person (from the LINNEAUS collaborative) in each of the countries and asked him or her to provide us with the names of 10 practising primary care physicians with a potential interest in patient safety and 10 researchers or experts in patient safety in their country. All were e-mailed and they received an invitation to the survey using an internet survey software programme. Non-respondents were sent a second invitation after one week and a third invitation one month later. The Medical Ethics Committee of the Radboud University Nijmegen Medical Centre approved this study.

### Questionnaire

The content of the questionnaire was based on earlier studies which explored what 'patient safety' consists of in primary care [8-10]. In addition, five telephone interviews with international patient safety experts were conducted to develop this questionnaire. A set of the most salient points was then selected and put into a questionnaire, which was subsequently reviewed by three experts on patient safety in order to fine-tune the questions. The web-based survey comprehended five themes (practice facilities, patient safety management, communication and collaboration, generic conditions for patient safety and education on patient safety), which consisted of 38 patient safety promotion strategies (e.g. incident reporting, medication alerts, patient safety indicators, periodic medication review, training on patient safety or culture conditions). For each strategy, we inquired about current use in their own country (no, no but planned, yes <50% of GPs, yes >50% of GPs), and whether the strategy constituted a promising approach (yes very much, yes to some extent, partly yes/partly no, no probably not, no certainly not). The respondent could also provide comments per theme. Finally, we asked if any other promising approaches were seen, which had not been mentioned in our questionnaire. The data were entered into SPSS 16.0 for analysis. To examine the homogeneity across country samples, we used ANOVA tests to examine the differences of perceptions between countries.

## Results

A total of 109 individuals were identified through the key persons from the different countries (between 4 and 36 per country). The survey was completed by 58 individuals. Table 1 reports on their characteristics. Fifty-one had a medical training, of which 46 were practising general practitioners (GPs). Three had a social science background and the remaining four individuals did not mention their discipline. The 46 practising GPs worked in practices that were spread across rural areas, towns and cities. There was a wide spread in the number of patients per practice. Only two significant country differences were found regarding the six main themes. The 58 participants made 108 comments in response to the open questions, which consisted mostly of practice examples. These comments were not further analyzed. Tables 2 reports on the views on patient safety strategies. We will discuss the most salient findings below.

**Table 1:** Demographic characteristics

Gender	Male 43
	Female 11
	Unknown 4
Current professional discipline (more options possible)	Medicine 51
	GP 46
	General internist 1
	Other primary care physician 1
	Medical teacher 10
	Policy advisor 8
	Scientific researcher 16
	Other or unknown discipline 7

Country	Austria 3
	Denmark 5
	France 3
	Germany 9
	The Netherlands 16
	New Zealand 7
	Slovenia 5
	United Kingdom 10

Practice size, mean (SD)	7540 (16273)

Area of practice	Rural 14
	Town 10
	City 19
	Missing/not appreciable 15

**Table 2:** Views on importance and implementation of patient safety interventions

Facilities in the practice	% scored "very much important for patient safety"	Percentage ">50% present in country"
Computerised medical record system, which is adequately kept	82.3	82.7

Telephone facilitities that allow quick access to the practice, particularly for urgent health problems	70.7	82.7

Planned checks of safety of equipment, medication, and other facilities in the practice	69.0	53.8

Access to web-based clinical guidance tools in daily practice	68.0	57.6

Forms for reporting incidents available	67.9	28.3

Working agreements with pharmacists when problems arise with delivering medication e.g. alerts, interaction	67.3	46.2

Reminders and alerts regarding safety issues, which are integrated in the medical record system	61.5	43.1

Computerised decision support regarding medication safety in daily practice	60.8	44.0

Computerised decision support regarding test ordering in daily practice	47.1	13.7

**Patient safety management**	% scored "very much important for patient safety"	Percentage ">50% present in country"

Practice-based reporting and analysis of incidents (e.g. significant event audit)	74.5	19.2

Reporting and analysis of incidents in small educational groups (e.g. quality circles)	66.0	7.7

Measurement and feedback on safety culture in general practices	60.4	3.8

Nationwide or regional educational reporting system for incidents	57.7	11.5

Measurement and feedback on indicators for patient safety	57.7	5.7

Hygiene protocols and guidelines present	56.9	39.6

Campaigns to increase patients' and public awareness of patient safety in general practice	39.6	3.8

Periodic audits by an external inspection authority	38.5	13.5

Nationwide or regional incident reporting weeks	33.3	2.0

Surveys and other types of consultations of patients regarding safety incidents	0	3.8

**Communication and collaboration**	% scored "very much important for patient safety"	Percentage ">50% present in country"

Standards for record keeping (ICPC coding, electronic records)	75.0	62.3

Integrated medical records for communication with specialists and others	65.4	9.4

Structured formats for information on referral of patients	61.5	22.6

Electronic prescriptions and integrated medication overview in the records from the pharmacist	59.6	17.2

Periodic review of medication by pharmacists in patients who use dangerous (combinations of) medication	51.9	3.8

Comprehensive analysis of prescribing decisions in the pharmacy, using decision support systems	49.1	53.8

Patient-held medical records	41.2	13.2

**Generic conditions for patient safety**	% scored "very much important for patient safety"	Percentage ">50% present in country"

Culture and mentality which facilitates learning from incidents	73.6	9.6

Understanding of patient safety in health professionals, particularly regarding how it differs from complications of treatment	64.2	9.6

Workload is perceived as acceptable in general practice	52.9	13.5

Adequate procedures for identifying and managing burn-out in health professionals	50.9	0

Availability of information technology in general practice, and skills to use these adequately	0	34.6

**Education on patient safety**	% scored "very much important for patient safety"	Percentage ">50% present in country"

Education on patient safety in the vocational training of GPs	81.1	23.5

A guideline on patient safety is available	80.9	15.2

Education on patient safety in the vocational training of practice nurses	79.2	8.9

Postgraduate education on patient safety of GPs	78.7	13.7

Postgraduate education on patient safety of practice nurses	77.1	7.0

Education on patient safety in the medical curriculum, before graduation	73.6	17.3

Education on patient safety in the nursing curriculum, before graduation	72.5	13.6

### Practice facilities

Most of the presented practice facilities were seen as important for patient safety. Highest ranked an up-to-date electronic medical record and good telephone access to the practice. Both items were reported to be widely present. Planned safety checks, access to web based clinical guidance tools, agreements with the pharmacist, electronic reminders and alerts and computerized medication decision support were ranked highly relevant by 60 to 70% of the participants. These items were also seen as widely present. Computerized decision support regarding test ordering was ranked lowest. (table 2)

### Patient safety management

Practice-based incident reporting was seen as important, also in small educational groups. Measurement and feedback on patient safety indicators, and the presence of hygiene protocols *(a protocol with suggestions how to improve hygiene in a practice) *also scored above average. Nationwide incident reporting was perceived as less important, and incident reporting weeks were seen as even less important. Periodic audits by an external inspection authority were also considered to be relevant. None of the respondents saw patient consultation and patient reporting as very important for patient safety. Hygiene protocols were mostly present, although all other items (mostly regarding incident reporting) were hardly ever present. (table 2)

### Communication and collaboration

Standards for record keeping (ICPC coding) were seen as most relevant, moreover they were quite often present. Electronic prescriptions, periodic review of polypharmacy and decision support systems were seen as very important by approximately half of the respondents, however these items were much less present. Patient-held medical records scored lowest, yet about 40% of the respondents found this item of very relevance for patient safety. (table 2)

### Generic conditions for patient safety

A good culture and a mentality to learn from patient safety incidents was seen as most relevant, but was not very much present. An acceptable workload and prevention of burnout was seen as very important by approximately half of the respondents. Yet the presence of these measures was very low. Information technology was not seen as important, although to some extent this was indeed present. (table 2)

### Education on patient safety

Education was seen as the most important factor to improve patient safety. About 70% to 80% of the respondents found educational strategies to enhance patient safety to be very relevant. Highest ranked the education of GPs, but the education of other health care workers involved scored highly as well. Also, the presence of a specific patient safety guideline (a guideline that consists of different strategies and suggestions to improve patient safety in primary care) was perceived to be relevant. Education on patient safety was not widely provided. (table 2)

### Other items relevant for patient safety

Lastly we inquired if the respondents found any other items relevant for patient safety, which had not been mentioned in the questionnaire. Eight respondents mentioned additional items. The comments can be divided into a number of categories: more (media) coverage on patient safety, education, a practice/organization assessment tool, and overall healthcare culture improvement.

## Discussion

We undertook a web-based survey to identify important strategies to improve patient safety, for which a group of international experts on patient safety was consulted. Most of them were practising primary care physicians. Although the majority of the 38 presented strategies were seen as important by most of the participants, the use of those strategies in daily practice varied widely. Strategies that yielded the highest scores (>70%) regarding importance included a good medical record system, good telephone access, standards for record keeping, learning culture, vocational training on patient safety for GPs and availability of a patient safety guideline. We suggest that strategies which are seen as important, but have been poorly implemented are the most promising for further research and development [8,10].

As far as we know, this study is one of the first to map the most important patient safety improving strategies, seen by experts in different countries with a strong primary care system.

This study has some limitations, which are described in the limitations section below. Nevertheless, some interesting trends were observed. First, it was noticed that the most well-known and already most researched (and implemented) items, namely a decent electronic medical record (including ICPC coding, and alert overkill) [11] and telephone accessibility, were perceived to be highly important and to have been widely implemented. In many countries these items have received a lot of attention. Nevertheless, there still seem to be practices which do not have these features, so improving these items could be relevant [10,12].

On the other hand, incident reporting was only perceived to be highly important, if it was organised in the physician's own practice or regionally. National incident reporting systems (e.g. such as known in the UK) were regarded as less important. Apparently, people experience a threshold when it comes to reporting incidents nationwide, despite the higher number of reports received in the NHS system [13].

Another item is the involvement of patients in patient safety strategies: the participants in our survey did not indicate that this was highly important. It is possible that it is perceived to be too early to involve patients in patient safety strategies [14].

There is little correlation between the intention of a health care worker and the subsequent (improvement) behaviour [15]. We found that the respondents in this study actually ranked all given educational items strikingly high on relevance for patient safety, while the actual presence in the European countries was low. This suggests that education on patient safety in vocational training and postgraduate programmes is a promising strategy. Also, a patient safety programme as education for practices (such as a prospective risk analysis) could be useful as a patient safety improvement programme. This is our goal for the next period in the LINNEAUS collaborative. Obviously, a positive culture for patient safety was also seen as highly important, which is consistent with other literature [16,17].

### Limitations

The response rate for this study was acceptable, but selection bias cannot be ruled out. Due to the selection procedure used (through a contact person), it is likely that we asked the most experienced patient safety practising primary care physicians in the different countries, and patient safety experts, on their opinion. Most of the respondents were actually practising GPs (46/58), which can be seen as a potential bias. Other health care personnel, such as managers or policy makers, could have been asked as well. However, practising GPs are the ones who are most likely to have the most direct view of the field. In earlier studies we noticed that 'regular' practising GPs found patient safety highly relevant, yet they had a very broad idea about patient safety. It is likely that GPs who are somewhat more experienced on patient safety will come up with better ideas to improve patient safety [8]. While the survey used in this study has not been empirically validated due to time restraints (through a Delphi procedure), it was nevertheless based upon the results of previous research [8-10] and interviews and the insights of experienced GPs with regard to the choice of clinical cases and potential risk factors [8-10,18]. Moreover, in order to develop this survey, the items were derived from interviews held with five experts on patient safety.

### Implications for future research

This study highlights the strategies that are seen as promising for the improvement of patient safety in primary care. Obviously, the effectiveness, efficiency and feasibility of these strategies have yet to be tested in well-designed evaluations. Possibly the most promising approach to improve patient safety (highly important and poorly implemented) is education for health professionals on patient safety. Therefore the need to develop educational tools, such as a prospective risk analysis for a practice, [19] specific guidelines on important patient safety features, or more attention on patient safety in the vocational training of primary care workers, seems a promising approach to improve patient safety. Until now, such a tool has not been present to our knowledge. Our goal in the next phase of the LINNEAUS program is to develop a web-based educational tool on patient safety.

## Conclusions

An international panel of primary care physicians and researchers felt that many different strategies to improve patient safety were important. Highly important strategies with poor implementation were a culture that is positive for patient safety, education on patient safety for physicians, and the presence of a patient safety guideline. The most promising patient safety implementation programs should focus on these items, in order to yield the best results.

## Competing interests

The authors declare that they have no competing interests.

## Authors' contributions

SG, WV and MW developed the study, SG collected the data, performed the analyses, presented the results and drafted the manuscript. WV contributed to the conception and design of the study and critically revised the manuscript. MW supervised the study, contributed to the conception and design of the study and critically revised the manuscript. All authors read and approved the final manuscript.

## Pre-publication history

The pre-publication history for this paper can be accessed here:

http://www.biomedcentral.com/1472-6963/11/102/prepub
